# Dr. George Watt

**Published:** 1893-04

**Authors:** 


### Obituary.

### DR. GEORGE WATT.

Through the kindness of the publishers of the *Ohio Dental Journal*, we are enabled to give abstracts of the work of the life of Dr. Watt, prepared by Dr. J. Taft for that journal in 1888.

"Died, at Xenia, Ohio, February 16, 1893, of locomotor ataxia, Dr. George Watt, M.D., D.D.S., in the seventy-third year of his age.

"Dr. George Watt was born on March 14, 1820, on a farm eight miles east of Xenia, Greene County, Ohio. His father, Hugh Watt, was born in the north of Ireland of Scotch parentage; his mother was a native of Western Pennsylvania, and was also of Scotch parentage.

"He first entered a country school at seven years of age. Schools at that time were very primitive and were generally continued only for three or four months during the year. He remained at the family home till October, 1835, when he left and went to Adams County, Ohio, to enter a boys' academy, established and conducted by Rev. William Taylor.

"In 1840 he entered a college at Ripley, Brown County, Ohio, and remained about a year; leaving there in 1841, he returned to Greene County, and there engaged in teaching and the study of medicine, in which he had the late Dr. Samuel Martin, of Xenia, for his preceptor, with whom he not only had superior instruction in medical science and practice, but also had there inculcated, in a receptive mind, the views of the dignity and importance of medical science and practice that have ever characterized him from that time to the present.

"He practised with and for his preceptor about one year, after which he removed to Bentonville, a small town in Fayette County, Indiana, where he soon established an excellent practice, especially for a new country. Not being satisfied with his attainments in medical science, he entered the Medical College of Ohio in 1846, and graduated in March, 1848.

"April 17, 1845, he was married to Miss Sarah Jane McConnell, who was a native of Greene County, Ohio.

"Early in the year 1852 he entered upon the study of dentistry. He was well prepared for such a course of study by his medical knowledge. After some time devoted to this specialty, he formed a co-partnership with J. Taft, of Xenia, Ohio, for the practice of dentistry, which continued for many years. One branch of science to which Dr. Watt had given much attention during and after his medical course was chemistry. Some idea of his proficiency in this direction will be apparent when it is remembered that he had an unusual aptitude in and liking for this branch, and also that he had for his preceptor that noted chemist and philosopher, the late lamented Professor John Locke, who was for many years a teacher in the Medical College of Ohio. In 1853 he prepared and delivered a course of lectures on chemistry in the Ohio College of Dental Surgery. And though Dr. Elijah Slack, M.D., LL.D., had delivered a course of lectures on chemistry in the same institution for one or two years before, yet this effort of Dr. Watt was the first attempt to adapt a course of lectures on chemistry to the need of the dental students, and he was the first to prepare and deliver such a course. All work in this line done before him was simply such as was given to medical students. During the time in which this course was delivered he was not only a teacher hewing out a new course for himself, but he was a member of the class he taught, so that he was in the double capacity of a teacher and a pupil.

"He graduated with his class, receiving the degree of Doctor of Dental Surgery at the close of the term of 1854.

"Immediately after this he resumed the practice of dentistry with Dr. Taft. So well recognized was his ability as a teacher that he was not long permitted to enjoy the quiet and comfort of a town and country practice, for in the spring of 1855 he was elected Professor of Chemistry and Metallurgy in the Ohio College of Dental Surgery, which position he occupied for several years.

"He became a member of the Mississippi Valley Dental Society in 1852.

"He, with Dr. Taft, became the owner and editor of the *Dental Register of the West.* This relation was maintained for many years, and was only relinquished when Dr. Watt's failing health rendered it imperative. But he has always, and even after this severance, been a liberal contributor to the literature of the profession.

"In the autumn of 1860, in the time of the great trial of our nation, he promptly tendered his services, so far as possible, for its rescue; however, he was not accepted until May, 1864, when he was made surgeon of the One Hundred and Fifty-fourth Regiment of Ohio Volunteer Infantry.

"His efficiency in that position will be well understood when it is stated that the sanitary record of his charge was better than that of any other Ohio regiment.

"He was mustered out September, 1864, after being disabled by an injury to the spine, having been crushed by a falling wagon, which resulted in locomotor ataxia.

"After his return from the army he entered into practice, as his feeble health would permit.

"In the autumn of 1865 he formed a partnership with Dr. N. W. Williams, after which they conducted a practice in Xenia for about one year, and they established a branch office in Cincinnati, of which Dr. Watt took charge.

"In 1868 the firm of Watt & Williams purchased the dental depot of J. S. Walters & Co., of Cincinnati. This business they conducted successfully, in addition to their dental practice, for about three years, when they sold the depot to Spencer & Moore.

"During this time his health was becoming more feeble, and his strength at times seemed well-nigh gone, and it was deemed best that he should retire and no longer attempt the active duties of practice. But he was not content to be idle. In 1881 he took the editorship of the *Ohio Journal of Dental Science*, a monthly journal of about fifty pages.

"In 1867 he published a volume entitled 'Watt's Chemical Essays,' in which are published the principal papers which he has written on dental chemistry.

"Quite a number of these papers have been published in various journals, not only of the dental but of the medical profession as well, and even in some of the leading newspapers of the country. Unfortunately, the syllabus of his series of lectures on chemistry has never been published.

"He has occupied a number of positions of prominence in addition to those already named. He was Vice-President of the American Dental Convention. He was elected President of the American Dental Association on the same day be became a member, the only instance of the kind on record. He was President of the Ohio State Dental Society the first two years of its existence; was twice President of the Mad River Dental Society. In every responsible position he ever occupied the duties devolving on him were discharged faithfully and efficiently."

His widow, Mrs. S. J. Watt, and their adopted daughter, Mrs. Sillito, are the only survivors of his immediate family.

The funeral services were held at his late residence on Saturday morning, February 18.

